# Prevalence of babesiosis in bovines of India: a meta-analytical approach for 30 years (1990–2019)

**DOI:** 10.1080/01652176.2023.2185695

**Published:** 2023-03-14

**Authors:** Udipta Borthakur, Med Ram Verma, Yash Pal Singh, Sanjay Kumar, Dinesh Kumar, Yogesh Chandrakant Bangar, Khan Sharun, Kuldeep Dhama

**Affiliations:** a Division of Livestock Economics, Statistics and Information Technology, ICAR-Indian Veterinary Research Institute, Bareilly, Uttar Pradesh, India; b Department of Animal Genetics and Breeding, The Lala Lajpat Rai University of Veterinary and Animal Sciences (LUVAS), Hisar, Haryana, India; c Division of Surgery, ICAR-Indian Veterinary Research Institute, Bareilly, Uttar Pradesh, India; d Division of Pathology, ICAR-Indian Veterinary Research Institute, Bareilly, Uttar Pradesh, India

**Keywords:** Cow, cattle, bovine, buffalo, babesiosis, India, meta-analysis, prevalence, systematic review

## Abstract

**Background:**

India has a massive population of bovines, which makes the framework of the economy mainly relying on milk and meat production. Parasitic diseases such as babesiosis are detrimental to bovines by decreasing animal welfare and production efficiency.

**Aim:**

Performing a meta-analysis of the prevalence of babesiosis over 30 years viz 1990 to 2019 within India to pool out individual studies from different country regions.

**Material and methods:**

The studies were reviewed thoroughly to assess the quality, and it was done by following the preferred reporting items for systematic review and meta-analysis (PRISMA) and MOOSE protocols. The prevalence of babesiosis in cattle and buffaloes was calculated using meta-analysis tools using R-software and Q Statistics.

**Results:**

The systematic review and meta-analysis performed on 47 studies among bovine, 48 studies among cattle, and 13 studies among buffaloes revealed the (pooled) prevalence of babesiosis in India as 10.9% (6.3%–18.2%; *Q* = 5132.03, d.f. = 46, *P* < 0.001), 11.9% (6.9%–19.8%; *Q* = 5060.2, d.f.=47, *P* < 0.001), and 6.0% (2.6%–13.2%; *Q* = 500.55, d.f.=12, *P* < 0.001), respectively, which provides a rather exact scenario of the prevalence of this haemoparasitic disease across the country. In addition, cattle were having higher risk of babesiosis than buffalo.

**Conclusion:**

The findings from the meta-analysis showed that the disease is prevalent across the country, and that bovines are highly affected by it.

**Clinical relevance:**

Appropriate prevention and control measures should be taken to mitigate this disease and enhance welfare and production performances of bovines.

## 1. Introduction

In India, since the ancient era, livestock has played a pivotal role in agriculture. For doubling the farmers’ income, livestock has immense importance out of which the bovines, especially cattle and buffaloes, serve the key role to the economy among the livestock. India is the highest livestock owner with diversified genera of breeds of livestock and poultry, which have a pivotal role in the socio-economic development of rural households. The livestock and agriculture sectors are intrinsically linked to producing the consumables and ensuring the food security of the nation.

Several diseases cause a negative impact on livestock health and production as well as have high economical importance (Perry and Grace 2009; Dhama et al. 2014). The consequences of animal diseases in livestock are detrimental and cause producers to lose in terms of production, resources, and maintenance. Parasitic diseases of bovines like babesiosis, an infectious tick-borne haemoprotozoal disease caused by *Babesia bigemina* and *Babesia bovis*, can greatly affect the animal’s health status leading to high economic losses and is among the most prevalent and costly tick-borne diseases (TBD’s) of cattle worldwide (Jacob et al. 2020; He et al. 2021). Other babesia species affecting bovids include *B. orientalis*, *B. ovata*, *B. major*, *B. motasi*, *B. U sp. Kashi* and *B. venatorum*. The disease is also termed as bovine babesiosis, piroplasmosis, Texas fever, redwater fever, tick fever, or Tristeza (Zaugg 2009). It was first detected by Babes in 1888. It is common in tropical and sub-tropical regions worldwide with relatively high morbidity and mortality rate, and efforts are being carried out with regards to prevention and control measures (Suarez and Noh 2011; Gohil et al. 2013; Jacob et al. 2020; He et al. 2021). The disease is mechanically transmitted by ticks, in bovines, mainly by the genera of *Boophilus* and *Hyalomma* spp. (Ravindran et al. 2002).

Cattle acquire the infection by the introduction of the sporozoite stage of the parasite into the bloodstream from an infected tick during a blood meal (Barman et al. 2018). It has high economic importance as India annually loses around 57.2 million USD in the livestock sector due to this disease. In a case study conducted on an organized farm in Meghalaya in 2012, it has been reported that due to *B. bigemina* infection in a milch cross-bred cow, a total of 51.6 liters loss of milk and an economic loss worth 1032 rupees (12.92 US dollar) due to decrease in production for 30 days (Laha et al. 2012).

Meta-analysis is a statistical technique that combines the results of several related studies over a topic and offers more accurate and reliable information on the effects of certain factors and treatments. In simple language, meta-analysis can be said an analysis of analysis. Meta-analysis of the prevalence of babesiosis in bovine will be useful to obtain pooled estimates of summary estimates from the published studies. It will help to better understand the prevalence of the disease associated with various risk factors. Therefore, the present study was carried out to estimate the prevalence of bovine babesiosis in India by performing a systematic review and meta-analysis compiling the data of 30 years (1990 to 2019).

## 2. Materials and methods

**Figure 1.**
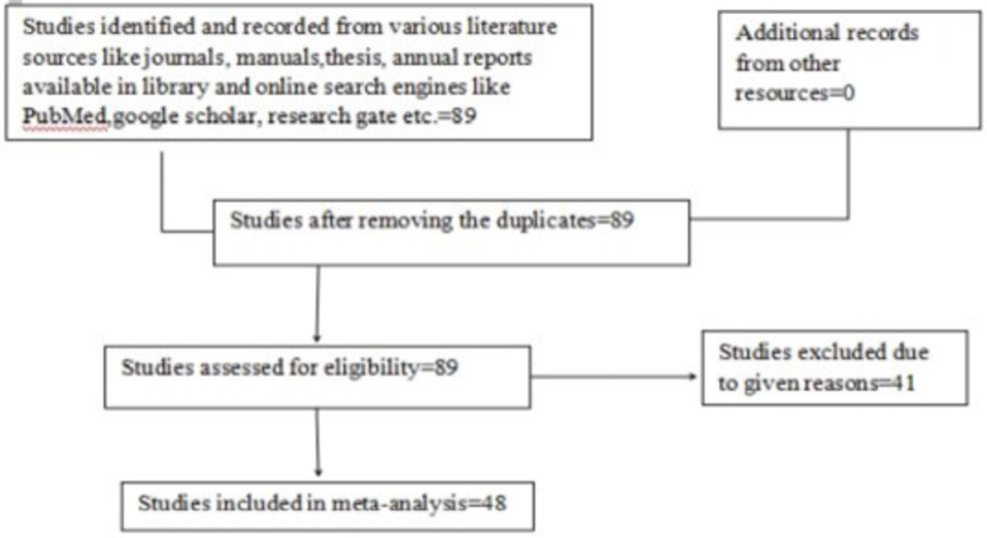
Flow diagram of study selection for meta-analysis of babesiosis in bovine in India respectively, and the outliers can be differentiated.

The systematic review of prevalence and risk factors of babesiosis in bovines was done from 1990 to 2019. Data collected from all the farms were clubbed with published studies to get a pooled estimate. Published studies were collected from various journals, annual reports, and online search engines like PubMed, Science Direct, Google scholar, NCBI, J-Gate, Krishikosh, etc. The studies were reviewed thoroughly to assess the quality, and it was done by following the preferred reporting items for systematic review and meta-analysis (PRISMA) and MOOSE protocols. Accordingly, the inclusion and exclusion criteria for studies were prepared and shown in Table 1. Flow diagram of study selection for meta-analysis of babesiosis in bovine is shown in Figure 1.

**Table 1.** Inclusion and exclusion criteria used in the study.

Inclusion criteria	Exclusion criteria
Studies included specified for the taken diseases, i.e. babesiosis.	Studies other than the mentioned diseases
Studies included only about bovines	Studies other than bovines
Studies specified to India only	Study radius outside India
Studies which were having a definite sample size	Studies having an indefinite or inadequate sample size
Random sampling	Purposive sampling or non-random sampling if tried
Publication Years (1990-2019)	Studies other than the said period
Studies depicted only the prevalence of the diseases of interest which were naturally occurred in an area or state in a given period.	Experimental studies or the studies where experimentally causing the disease to conduct clinical trials.

The prevalence of babesiosis in cattle and buffaloes was calculated using meta-analysis tools using R-software, with 48 published studies across India from 1990 to 2019 after screening and quality assessments. These again were subdivided into cattle and buffalo separately and subsequently 48 and 13 studies were included, respectively. The list of studies included in the meta-analysis of babesiosis is given in Table 2.

**Table 2.** Details of studies included in the meta-analysis of babesiosis.

Sl. no.	Author	Year	State	Diagnostic test used
1.	Chandra and Rajkhowa,1990	1990	Meghalaya	Microscopic (blood smear)
2.	Ansar Kamran et al. 1991	1991	Karnataka	Microscopic (blood smear)
3.	Shastri et al. 1991	1991	Maharashtra	Microscopic (blood smear)
4	Jithendran 1997	1997	Himachal Pradesh	Microscopic (blood smear)
5.	Mishra et al. 1998	1998	Uttar Pradesh, Orissa, Rajasthan	Microscopic (blood smear)
6.	Sharma et al. 2000	2000	Himachal Pradesh	Microscopic (blood smear)
7.	Bhikane et al. 2001	2001	Maharashtra	Microscopic (blood smear)
8.	Ravindran et al. 2002	2002	Kerala	Serological (IFAT)
9.	Agrawal et al. 2003	2003	Chhattisgarh	Microscopic (blood smear)
10.	Garg et al. 2004	2004	Uttarakhand	Microscopic (blood smear)
11.	Saud et al. 2005	2005	Arunachal Pradesh	Microscopic (blood smear)
12.	Aulakh et al. 2005	2005	Punjab	Microscopic (blood smear)
13.	Julie et al. 2005	2005	Kerala	Microscopic (blood smear)
14.	Muraleedharan et al. 2005	2005	Karnataka	Microscopic (blood smear)
15.	Harish et al. 2006	2006	Karnataka	Microscopic (blood smear)
16.	Singh et al. 2007a	2007	Uttar Pradesh	Serological (IFAT)
17.	Singh et al. 2007b	2007	Uttar Pradesh	Molecular (PCR)
18.	Ananda et al. 2009	2009	Karnataka	Microscopic (blood smear)
19.	Nair et al. 2011	2011	Kerala	Molecular (PCR)
20.	Rejitha and Devada 2011	2011	Kerala	Serological (IFAT)
21.	Singh et al. 2012	2012	Punjab	Microscopic (blood smear)
22.	Jyothisree et al. 2013	2013	Andhra Pradesh	Molecular (PCR)
23.	Singh et al. 2013	2013	Punjab	Molecular (PCR)
24.	Sharma et al. 2013	2013	Punjab	Molecular (PCR)
25.	Chaudhri et al. 2013	2013	Haryana	Microscopic (blood smear)
26.	Saravanan et al. 2013	2013	Arunachal Pradesh	Molecular (PCR)
27.	Ananda et al. 2014	2014	Karnataka	Microscopic (blood smear)
28.	Krishnamurthy et al. 2014	2014	Karnataka	Microscopic (blood smear)
29.	Velusamy et al. 2014	2014	Tamil Nadu	Microscopic (blood smear)
30.	Ananda et al. 2014	2014	Karnataka	Microscopic (blood smear)
31.	Kakati et al. 2015	2015	Assam	Microscopic (blood smear)
32.	Bhatnagar et al. 2015	2015	Rajasthan	Microscopic (blood smear)
33.	Sharma et al. 2016	2016	Punjab	Molecular (PCR)
34.	Kumar et al. 2016	2016	Gujarat	Microscopic (blood smear)
35.	Bal et al. 2016	2016	Punjab	Microscopic (blood smear)
36.	Maharana et al. 2016	2016	Gujarat	Microscopic (blood smear)
37.	Kaur et al. 2016	2016	Punjab	Serological (Indirect ELISA)
38.	Ganguly et al. 2017	2017	Haryana	Microscopic (blood smear) examination
39.	Bhat et al. 2017	2017	Punjab	Molecular (PCR)
40.	Kolte et al. 2017	2017	Maharashtra	Molecular (PCR)
41.	Vijayakumar et al. 2017	2017	Karnataka	Molecular (PCR)
42.	Ponnudurai et al. 2017	2017	Tamil Nadu	Microscopic (blood smear)
43.	Nimisha et al. 2017	2017	Kerala	Microscopic (blood smear)
44.	Kumar et al. 2018	2018	Bihar	Molecular (PCR)
45.	Barman et al. 2018	2018	Assam	Microscopic (blood smear)
46.	Gaurav et al. 2018	2018	Uttarakhand	Microscopic (blood smear)
47.	Maharana et al. 2018	2018	Haryana	Molecular (PCR)
48.	Durairajan and Murugan 2019	2019	Tamil Nadu	Microscopic (blood smear)

## 3. Results

### 3.1. Meta-analysis in cattle

**Figure 2.**
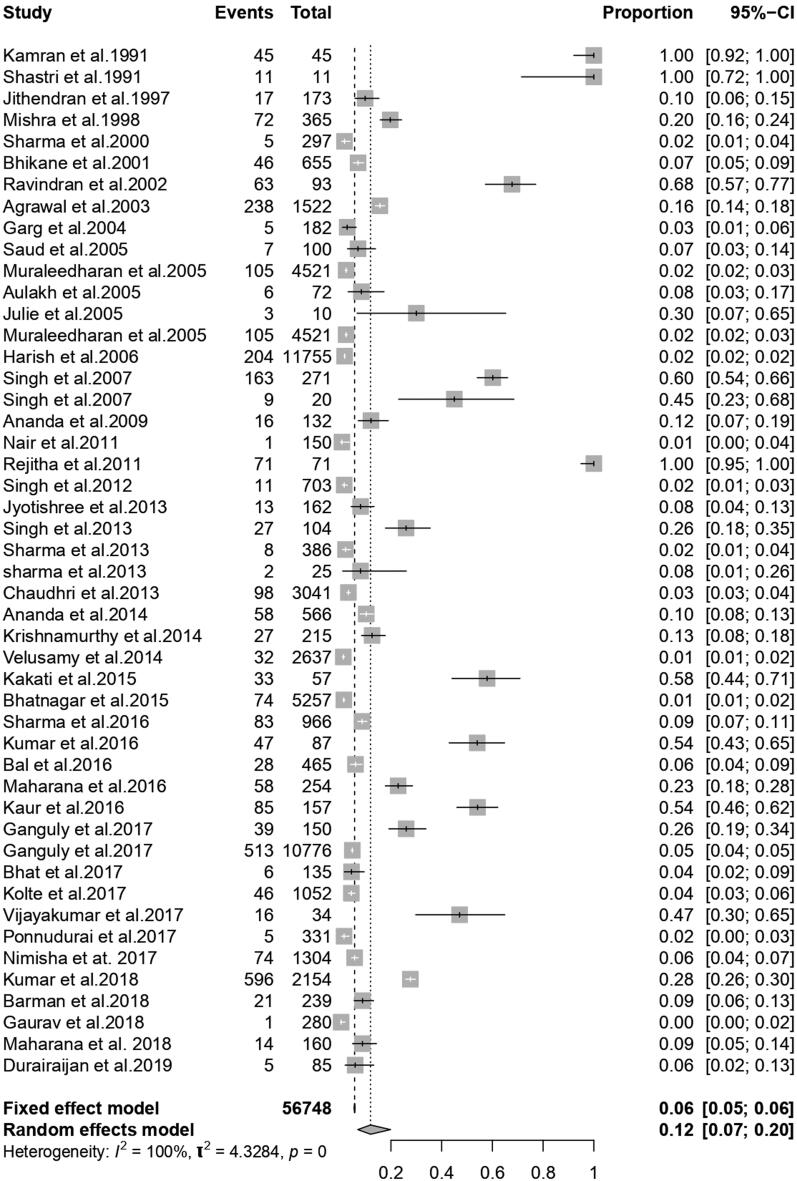
Forest plot showing studies reporting the prevalence of babesiosis in cattle in India.

Among the selected 48 published references, 56,748 cattle were considered for meta-analysis. Following the quality assessment, the pooled prevalence was found to be 11.9% (95% CI; 6.9; 19.8) with a significant Q statistics value (*Q* = 5060.2, d.f. = 47, *P* < 0.001). Variability between the studies was 4.3284 (tau-square), and the measure of heterogeneity was 99.5% (I^2^ Index). The forest plot (Figure 2) represents the proportion of cattle affected by babesiosis per individual studies and the overall pooled estimate of the prevalence of the disease.

### 3.2. Meta-analysis in buffaloes

**Figure 3.**
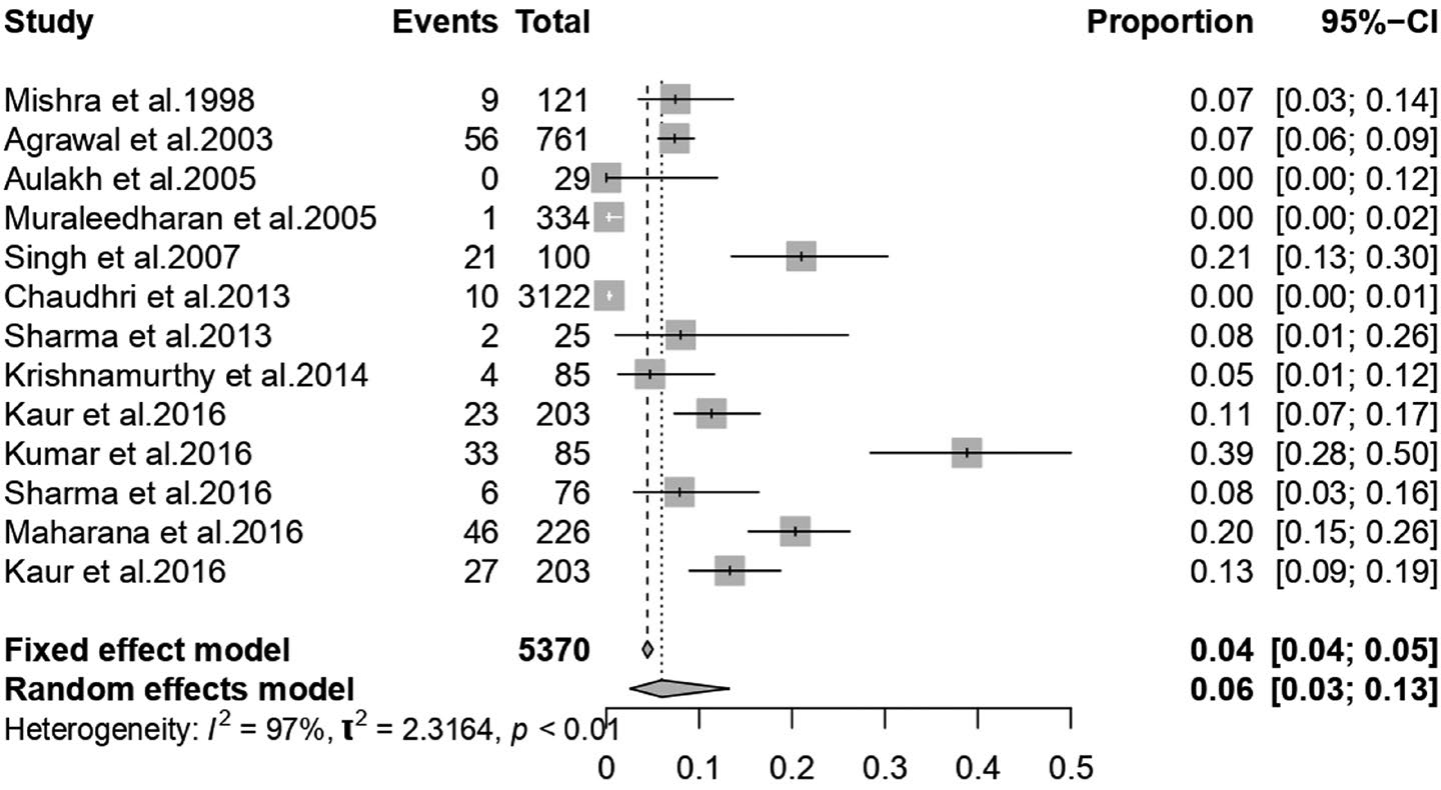
Forest plot showing studies reporting the prevalence of babesiosis in buffaloes in India.

From 13 published literature, 5370 cattle were included for the following meta-analysis, after the quality assessment and the pooled prevalence was found 6.0% (95% CI; 2.6; 13.2) with significant Q value (*Q* = 500.55, d.f.=12, *P* < 0.001), which stated that there was significant heterogeneity in between the 13 published studies. Variability between the studies was 2.3154 (tau-square), and the measure of heterogeneity was 97.1% (I^2^ Index). The forest plot (Figure 3) represents the proportion of buffalo affected by babesiosis per individual studies and the overall pooled estimate of the prevalence of the disease.

### 3.3. Meta-analysis in bovines

**Figure 4.**
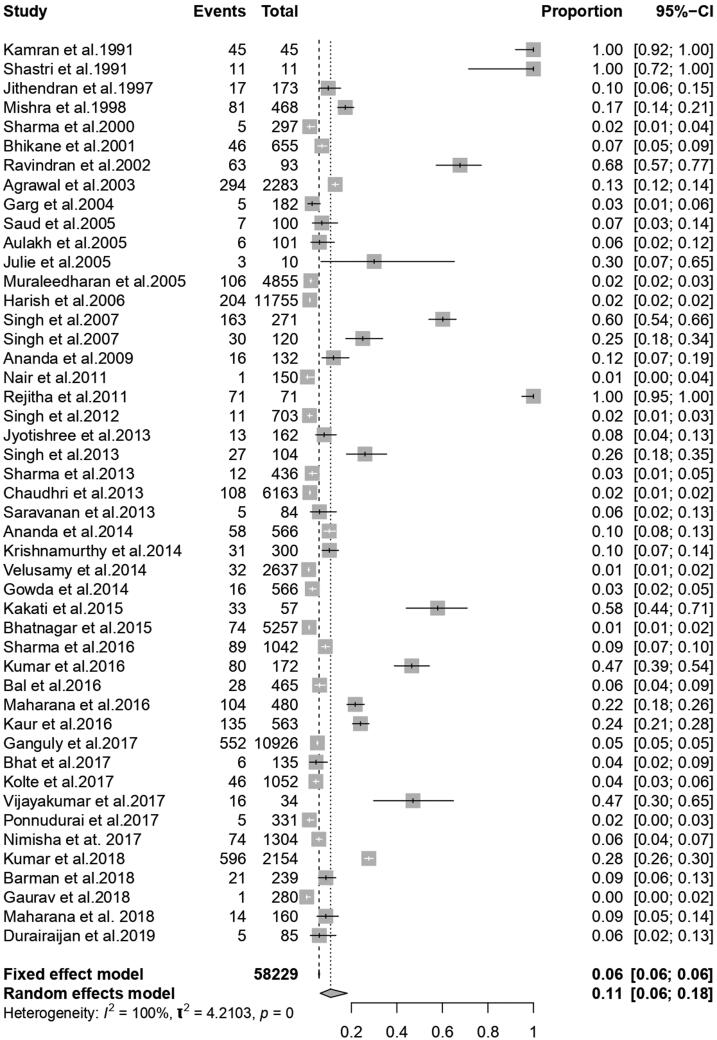
Forest plot showing studies reporting the prevalence of babesiosis in bovines in India.

From 47 published literature, 58,299 cattle were included for the following meta-analysis, after the quality assessment and pooled prevalence were found 10.9% (95% CI; 6.3; 18.2) whereas Q statistics were found significant (*Q* = 5132.03, d.f. = 46, *P* < 0.001). Variability between the studies was 2.0519 (tau-square) whereas the measure of heterogeneity was 99.6% (I^2^ Index). The forest plot (Figure 4) represents the proportion of bovines affected by babesiosis per individual studies and the overall pooled estimate of the prevalence of the disease.

### 3.4. Identification of publication bias in meta-analysis of the prevalence of babesiosis

**Figure 5.**
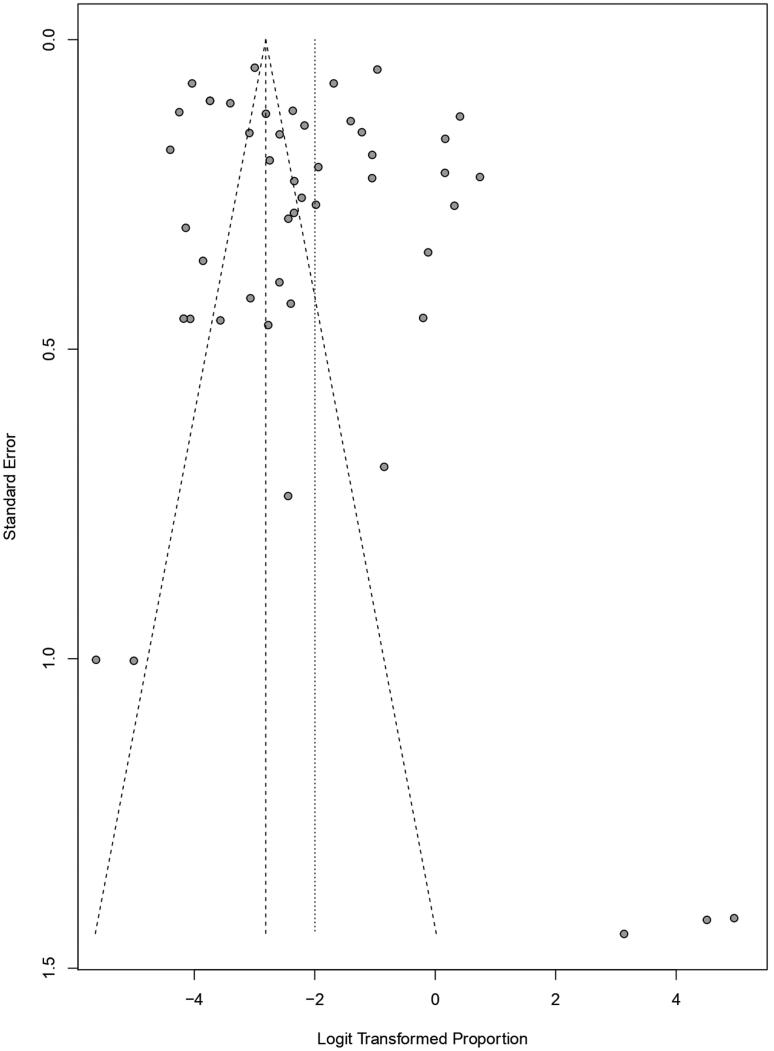
Funnel plot for identification of publication bias in meta-analysis of the prevalence of babesiosis among cattle.

**Figure 6.**
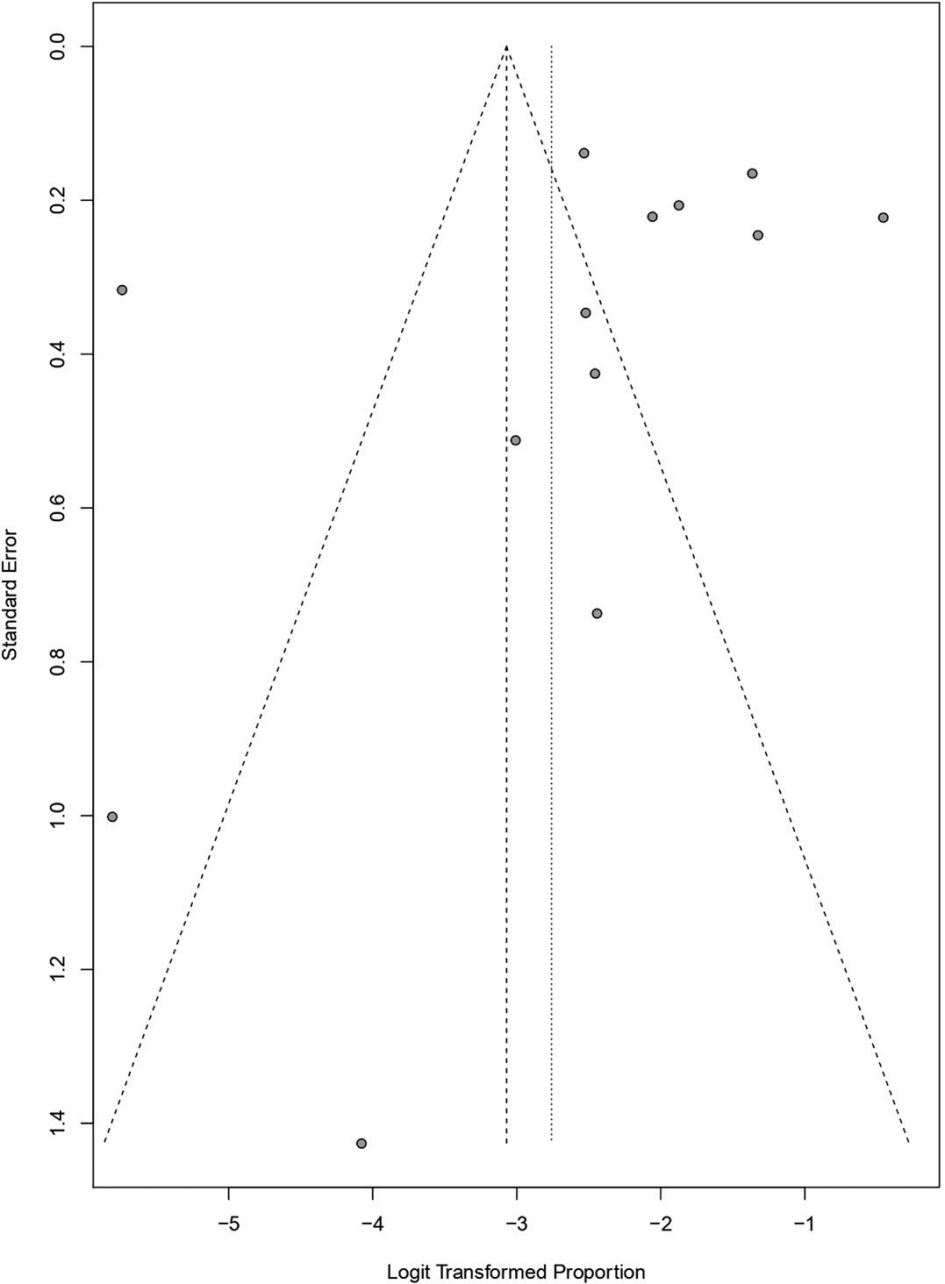
Funnel plot for identification of publication bias in meta-analysis of the prevalence of babesiosis among buffaloes.

**Figure 7.**
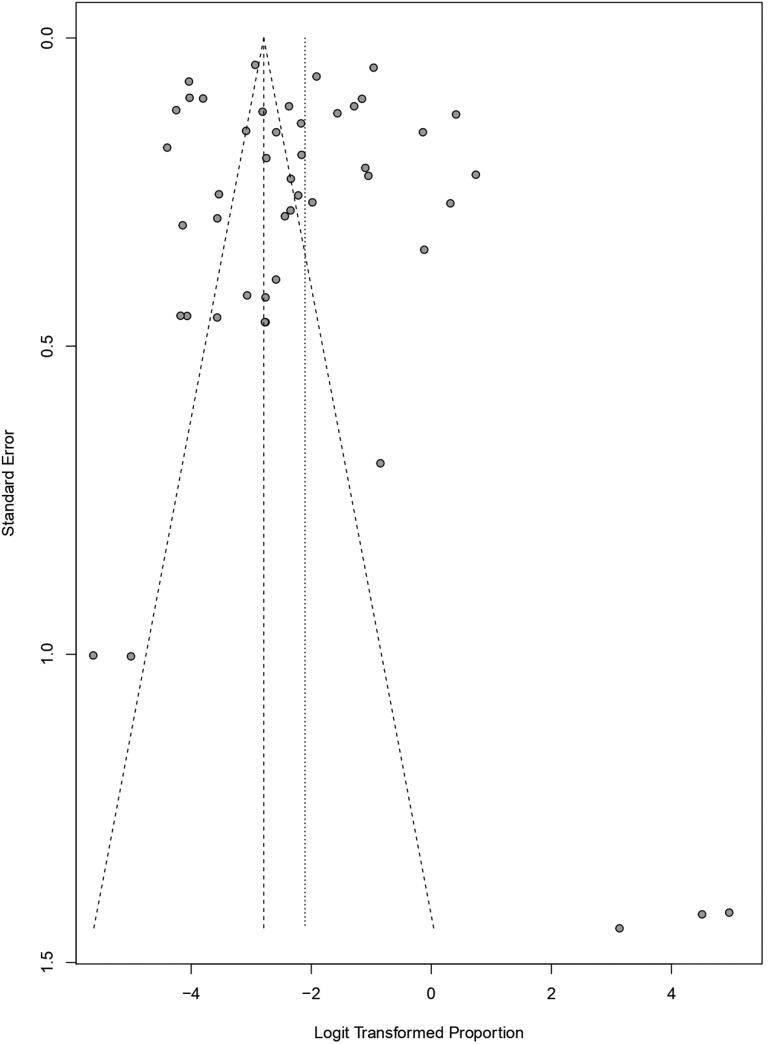
Funnel plot for identification of publication bias in meta-analysis of the prevalence of babesiosis among bovine.

Funnel plot techniques were used to identify the publication bias among the studies where the proportion of each study was plotted on the horizontal axis, while the standard error was on the vertical one. Figures 5, 6, and  7 show the funnel plots for studies taken for babesiosis in cattle, buffaloes, and bovine, respectively, where only a few studies were inside the funnel. In contrast, most of the studies were scattered outside of it, which implied significant publication bias. In addition, the studies are distributed scattered throughout the graph, which implies they have different standard errors.

## 4. Discussion

The present meta-analysis was performed to review prevalence of babesiosis in cattle, buffaloes and bovine in last 30 years in order to consider maximum number of studies for better conclusions.

Meta-analysis of the prevalence of babesiosis in cattle cited the pooled prevalence was 11.9% (6.9%–19.8%) which was related to the studies done by Ananda et al. (2009), who claimed the prevalence of babesiosis in cattle was about 12.1%. On the other hand, the meta-analysis in terms of buffaloes resulted that the pooled prevalence was 6.0% (2.6%–13.2%) which was found similar to the findings of Agrawal et al. (2003), who stated the prevalence was 7.4% along with other authors like Mishra et al. (1998); Sharma et al. (2013); Krishnamurthy et al. (2014) and Sharma et al. (2016) who also found similar results on the prevalence of babesiosis in buffaloes were 7.4%, 8%, 4.7%, and 7.9% respectively.

Meta-analysis of Babesiosis in bovines resulted in the pooled prevalence estimated as 10.9% (6.3%–18.2%), which was in accordance with Krishnamurthy et al. (2014), who stated that the prevalence of babesiosis in bovines was 10.3% along with the findings of authors, Jithendran (1997), Agrawal et al. (2003), Ananda et al. (2009), Ananda et al. (2014), and Sharma et al. (2016) who stated that the prevalence was 9.8%, 12.9%, 12.1%, 10.2%, and 8.5% respectively.

Meta-analysis of risk of babesiosis in cattle with respect to buffaloes was studied, which showed that cattle were having higher risk of babesiosis than buffalo. Similar findings were observed in many studies reported in India (Agrawal et al. 2003; Aulakh et al. 2005; Muraleedharan et al. 2005; Krishnamurthy et al. 2014; Kaur et al. 2016; Maharana et al. 2016).

## 5. Conclusion

The systematic review and meta-analysis analysed 47 studies among bovine, 48 studies among cattle, and 13 studies among buffaloes to estimate the prevalence of Babesiosis in India. The findings indicate a pooled prevalence of 10.9% (6.3%–18.2%), 11.9% (6.9%–19.8%), and 6.0% (2.6%–13.2%) in bovine, cattle and buffaloes, respectively. The climatic conditions, topography of the country enhance the growth and transmission of the vector, ultimately affecting the occurrence of the disease. The findings from the meta-analysis showed that the disease is prevalent across the country, and the bovines are highly affected by it. So, to mitigate the disease, appropriate prevention and control measures should be taken to safeguard bovine health and enhance production.

## Data Availability

The data that support the findings of this study are available from the corresponding author upon reasonable request.
